# The business case for hospital mobility programs in the veterans health care system: Results from multi‐hospital implementation of the STRIDE program

**DOI:** 10.1111/1475-6773.14307

**Published:** 2024-04-17

**Authors:** Brystana G. Kaufman, S. Nicole Hastings, Cassie Meyer, Karen M. Stechuchak, Ashley Choate, Kasey Decosimo, Caitlin Sullivan, Virginia Wang, Kelli D. Allen, Courtney H. Van Houtven

**Affiliations:** ^1^ Center of Innovation to Accelerate Discovery and Practice Transformation (ADAPT) Durham VA Medical Center Durham North Carolina USA; ^2^ Population Health Sciences Duke University School of Medicine Durham North Carolina USA; ^3^ Duke Margolis Institute for Health Policy Duke University Durham North Carolina USA; ^4^ Department of Medicine Duke University Durham North Carolina USA; ^5^ Department of Medicine University of North Carolina Chapel Hill North Carolina USA

**Keywords:** cost of care, geriatrics, implementation science, veterans

## Abstract

**Objective:**

To conduct a business case analysis for Department of Veterans Affairs (VA) program STRIDE (A**S**sis**T**ed Ea**R**ly Mob**I**lization for hospitalize**D** older V**E**terans), which was designed to address immobility for hospitalized older adults.

**Data Sources and Study Setting:**

This was a secondary analysis of primary data from a VA 8‐hospital implementation trial conducted by the Function and Independence Quality Enhancement Research Initiative (QUERI). In partnership with VA operational partners, we estimated resources needed for program delivery in and out of the VA as well as national implementation facilitation in the VA. A scenario analysis using wage data from the Bureau of Labor Statistics informs implementation decisions outside the VA.

**Study Design:**

This budget impact analysis compared delivery and implementation costs for two implementation strategies (Replicating Effective Programs [REP]+CONNECT and REP‐only). To simulate national budget scenarios for implementation, we estimated the number of eligible hospitalizations nationally and varied key parameters (e.g., enrollment rates) to evaluate the impact of uncertainty.

**Data Collection:**

Personnel time and implementation outcomes were collected from hospitals (2017–2019). Hospital average daily census and wage data were estimated as of 2022 to improve relevance to future implementation.

**Principal Findings:**

Average implementation costs were $9450 for REP+CONNECT and $5622 for REP‐only; average program delivery costs were less than $30 per participant in both VA and non‐VA hospital settings. Number of walks had the most impact on delivery costs and ranged from 1 to 5 walks per participant. In sensitivity analyses, cost increased to $35 per participant if a physical therapist assistant conducts the walks. Among study hospitals, mean enrollment rates were higher among the REP+CONNECT hospitals (12%) than the REP‐only hospitals (4%) and VA implementation costs ranged from $66 to $100 per enrolled.

**Conclusions:**

STRIDE is a low‐cost intervention, and program participation has the biggest impact on the resources needed for delivering STRIDE.

**Trial Registration:**

ClinicalsTrials.gov NCT03300336. Prospectively registered on 3 October 2017.

**What is known on this topic**

- Hospital mobility programs can reduce rates of hospital‐associated disability, but they are not widely available.
- A Department of Veterans Affairs (VA) program called STRIDE (A**S**sis**T**ed Ea**R**ly Mob**I**lization for hospitalize**D** older V**E**terans) was designed to address immobility using supervised walks for hospitalized older adults.
- In prior evaluations, STRIDE was found to reduce odds of discharge to a Skilled Nursing Facility (SNF).

**What this study adds**

- This budget impact analysis informs the business case for disseminating STRIDE in and out of the VA.
- STRIDE is a low‐cost intervention to deliver, and program participation among eligible veterans has the biggest impact on the resources needed for delivering STRIDE.
- Achieving improved veteran outcomes through national STRIDE dissemination may require substantial investment of VA resources to facilitate successful implementation.

## 1 INTRODUCTION

Acute medical illness that requires hospitalization is common among older adults with chronic conditions, and disability is common following hospitalization in and out of the U.S. Veterans Health Administration (VHA).^1^, ^2^ Functional disabilities acquired during hospitalization impact the ability to conduct activities of daily living following hospitalization and may contribute to a need for nursing home care.^3^ Hospital mobility programs can reduce rates of hospital‐associated disability, but they are not widely available, and little is known about the cost of initiating hospital mobility programs in and out of the VA. Business case analyses in the context of implementation science studies can help provide a realistic picture of how much program delivery and implementation would cost for administrators seeking to initiate a similar hospital mobility program.

A Department of Veterans Affairs (VA) program called STRIDE (A**S**sis**T**ed Ea**R**ly Mob**I**lization for hospitalize**D** older V**E**terans) was designed to address immobility using supervised walks for older adults in VA hospitals. STRIDE provides hospitalized Veterans with a targeted gait and balance assessment by a physical therapist, followed by daily walks supervised by a mobility assistant for the remainder of their hospital stay. STRIDE was implemented as a part of the Optimizing Function and Independence Quality Enhancement Research Initiative (Function QUERI) in 2017–2019. In prior evaluations, STRIDE was found to increase discharge to home from 74% to 92% compared with skilled nursing facility (SNF) or other rehabilitation setting, consistent with findings from other similar hospital mobility programs.^4^, ^5^, ^6^

In this study, the trial evaluated two implementation strategies. All hospitals received support based on the Replicating Effective Programs (REP) framework, consisting of a series of activities to support implementation of the core elements of a program while allowing space for provider and staff input and flexibility to modify the program to hospital specific resources and patient needs.^7^ In addition to REP, four of eight participating hospitals were randomized to receive CONNECT, a complexity‐science‐based training to promote communication and multidisciplinary team collaboration.^8^, ^9^ CONNECT was adapted from a training originally called “CONNECT for quality,” which was evaluated as part of a falls prevention program in nursing homes.^10^

Outside the VA, increasing participation in value based payment models has heightened the need for economic evaluations to support the business case for upstream interventions, such as STRIDE, to prevent future adverse events and expenses. As the largest integrated health system, the VHA is motivated to accelerate adoption of evidence‐based programs and use limited resources efficiently to better serve veterans. Despite the differences in financial models between VA and non‐VA hospitals, the clinical staff roles, time and activities necessary to support a mobility program are similar in and out of the VA. Thus, the VA experience may provide useful insights for non‐VA health systems that have similar goals.

The purpose of this paper is to estimate the resources needed for STRIDE to be implemented more broadly in and out of the VA, using evidence from the 8‐hospital implementation trial.^4^, ^11^, ^12^ We present a base case scenario from the VHA perspective that highlights the individual hospital‐level costs and the total average costs across the hospitals. We distinguish costs of (1) the two implementation strategies tested in the STRIDE trial (REP + CONNECT and REP alone) and of (2) the program delivery. In addition to the base case estimates, we provide a range of likely cost outcomes if the program was implemented nationally based on the outcomes observed from the eight hospitals.^13^ This study addresses both implementation and delivery costs to promote the spread and scale of effective hospital mobility programs in and out of the VA.

## 2 METHODS

As a part of the larger Function QUERI study (Trial registration: ClinicalsTrials.gov NCT03300336), our team implemented STRIDE at eight VA medical centers nationally using two implementation strategies. Implementation costs were estimated for two implementation strategies: (1) REP, a facilitated implementation strategy (four hospitals) and (2) REP + CONNECT, a complexity‐science‐based training to promote communication and multidisciplinary team collaboration (four hospitals).^14^

To inform the business case for STRIDE, we conducted a budget impact analysis (BIA) to assess the resources needed to implement STRIDE in VA and non‐VA hospitals. Because STRIDE was previously shown to be effective in improving veteran outcomes, in the business case analysis, we assumed that implementation of STRIDE will lead to equivalent patient outcomes.^4^, ^6^, ^11^ We also assumed that patient outcomes will not differ at hospitals with staff members participating in CONNECT compared with REP‐only. First, we assessed the resources and associated costs at the eight hospitals that implemented the program during the study period. Second, we estimated the resources needed for national expansion of the program to inform VA decision‐making. Hospital‐level costs were estimated using a micro‐costing approach and the national projection base case was estimated using an average of hospital‐level costs.^15^ In addition, we conducted one‐way sensitivity analyses to illustrate the impact of each input parameter on total cost and a scenario analysis to assess the impact of reach rates for national implementation. Combined, these methods can show a realistic range of budget impacts from implementing a program like STRIDE.

As recommended for BIA, we built the analysis using a few key parameters, such as the prevalence of hospitalization, the percentage of those hospitalized who were evaluated and received STRIDE, and the cost of delivering STRIDE.^16^, ^17^ In this analysis, we assessed total program costs rather than incremental costs compared with another strategy because the intention was to assess resources needed in the context of a business case for implementation rather than a cost‐effectiveness evaluation.^18^ STRIDE was implemented at participating hospitals as a clinical program; evaluation was approved as human subjects research by the Durham VA Institutional Review Board.

## 3 STUDY POPULATION

STRIDE was designed for patients age 60 or older, the demographic group at highest risk for deconditioning and functional decline due to immobility in the hospital.^19^ This analysis uses data for all patients eligible and enrolled in STRIDE at the eight participating hospitals whose hospitalization(s) met inclusion and exclusion criteria for the parent study.^20^ There are those who may have received STRIDE who were not included in the analyses due to not meeting criteria (e.g., age <60, transferred from another hospital or nursing home, admitted to a nonmedical setting [e.g., surgery service]). To estimate the potential enrollment in the national analysis, we obtained the average daily census (ADC) for general medicine wards across all VA facilities nationally (ADC = 5235) for Fiscal Year 2022 Quarter 1. We also described facility complexity level to inform generalizability of the estimates. The VHA classifies facilities at levels 1a, 1b, 1c, 2, or 3 with level 1a being the most complex and level 3 being the least complex. The complexity algorithm is intended to categorize facilities into peer groups and is based on seven variables relating to patient population, clinical services complexity, and education and research.^21^

## 4 ASSESSMENT OF COSTS

Costs were assessed from the perspective of VA in adopting this program for clinical care. We did not consider the opportunity costs of staff time who may have shifted their resources from other duties to the STRIDE intervention tasks. That is, we assumed their time on STRIDE is revenue neutral to the health care system and that their time on STRIDE is what is relevant to the cost calculations. This BIA focused on the short‐term resources necessary for implementation, and costs were annualized to 1 year of delivery of STRIDE. Thus, no discounting was necessary due to the limited time horizon.

### 4.1 Program delivery activities and costs

The program consisted of a one‐time gait and balance assessment conducted by a physical therapist, followed by daily supervised walks led most commonly by a therapy or nursing assistant for the duration of the hospital stay. Patient identification included coordination with other providers, developing or conducting marketing and education materials, and other recruitment tasks. Walking included minutes (a) finding a patient to walk; (b) preparing the patient to walk; (c) walking with the patient; and (d) documenting the walk in the electronic health record. Miscellaneous tasks included capturing unsuccessful walk attempts, coordinating patient care and other non‐walking tasks such as triaging consults, and retrieving equipment such as assistive devices from storage. Walk time data were entered into standard STRIDE program electronic health record notes by clinical staff, and miscellaneous time data before and after the walk were captured by a time‐log survey (Appendix A, supporting information).

In providing the STRIDE program, participating hospitals incurred equipment and labor costs. Equipment costs included rolling walkers, tape measures, stop watches, pedometers, distance markers, portable pulse oximeters, gait belts, rollators, and canes as needed. Equipment costs were assessed using invoices, which were self‐reported by hospital Point of Contact to study team. Only one hospital purchased new equipment to implement STRIDE (Appendix B, supporting information); the other seven hospitals repurposed existing equipment. The invoiced expenses for the equipment averaged across hospitals were negligible and excluded from the analysis as sunk costs.

Labor costs were calculated as the time required to conduct patient identification, one‐time gait and balance assessments, and the supervised walks multiplied by the median salary for the providers conducting the activities. Providers delivering the service typically included the evaluating therapist (physical therapist) and the mobility assistant (physical therapy assistant or certified nursing assistant). Times were self‐reported to the study team via time‐logs conducted on a sample of 3–5 patients per facility. Time was assessed as the sum of reported time spent by providers during pre‐ and post‐walk activities as well as the time spent walking with patients from the electronic health record. Because the walk time was tracked in health factors as a categorical measure (1–5, 6–10, 11–15, 16–20, or >20 min), we estimated walk time as the midpoint value within each range; as walk time >20 min was open‐ended, we used a value of 20 for estimated walk time for that category based on time‐logs. For missing data for before or after walk time on time‐logs, the mean response from all the reporting hospitals was used. In estimating delivery costs, salaries are based on the state VA average geographic adjustment, not the specific locality to improve generalizability while still reflecting geographic variation in wage rates.

### 4.2 Implementation activities and costs

Implementation costs included labor for hospital personnel as well as the external support staff providing implementation support. A digital toolkit was also developed to support implementation simulated in this study and provided guidance on core and modifiable components including options for customization. Because the resources to develop these materials were a sunk cost (meaning expenses incurred prior to the decision point), we did not include these costs in the BIA for hospital or national projection.^15^, ^16^, ^17^, ^18^ While delivery costs were estimated using localized General Schedule (GS) payscale wages, implementation expenses were based on median national GS wages for the role (as of 2022); thus, all variation was driven by effort and not locality. This was necessary to support comparison of hospital resource use across implementation strategies. In estimating implementation costs, salaries for providers were assessed using median national base pay for each role and GS level. Adjustment for locality pay was accomplished by identifying and incorporating the median national locality adjustment (20.02%) across all hospitals.

### 4.3 REP activities that apply to both implementation strategies

*Prelaunch activities* were conducted under both implementation strategies and required both external staff and hospital staff resources. Virtual meetings comprised of five sessions that averaged between 60 and 75 min each covering topics such as introduction to STRIDE, program documentation, program marketing, hospital visit planning, and technical assistance calls. The 1‐day hospital visit was face‐to‐face and included implementation activities with the following personnel (Veterans Integrated Services Network [VISN]) chief medical officer, Chief of Geriatrics and Extended Care, physical therapists and supervisors, physical therapy assistants, Electronic health record coordinators/Clinical Applications Coordinators, hospitalists, fellows, rehabilitation coordinators, nurses, nurse managers, administrative officers, and data analysts. External (study team) personnel included study PI's, implementation specialists, project coordinators, qualitative analyst, research fellow, research assistants, statistician, and physical therapist.

*Postlaunch facilitated implementation support* was conducted under both implementation strategies and required both external staff and hospital staff resources. Postlaunch facilitated support centered around review of external‐generated workload reports and subsequent troubleshooting of identified inconsistencies. Virtual meetings occurred at Month 1 and Quarters 1–4 after launch. As in prelaunch support cadence, some hospitals opted to have additional touchpoints with the external team for technical assistance.

### 4.4 CONNECT activities that apply to the four REP+CONNECT hospitals

While REP activities largely involved people involved in implementation or delivery of STRIDE at each hospital, CONNECT targeted a larger group of hospital staff including all clinical personnel on inpatient units designated for STRIDE implementation, whether or not they had a specific role in implementing or delivering STRIDE. CONNECT activities included an added day to the hospital visit. The CONNECT training was delivered by masters‐level research staff using a standardized facilitator's guide and workbook.^10^

## 5 NATIONAL BUDGET PROJECTION AND SCENARIOS ANALYSES

In this intent‐to‐treat evaluation, all eight study hospitals were included regardless of implementation outcomes because excluding underperforming sites would bias the national projections and overstate the success of the program. We used the results of the hospital‐level BIA to project the potential resources needed for the national implementation of STRIDE at 60 hospitals. This trial collected data for eight hospitals implementing STRIDE; however, 59 VA hospitals with acute general medicine beds have planned to implement STRIDE by the end of Fiscal Year (FY) 2022. While 10 hospitals with less than 25 beds have planned to implement STRIDE, our cost data may not generalize to very small hospitals, and it is unclear whether different resources would be needed in this context. Thus, if STRIDE were mandated nationally after FY2022, we assumed national implementation costs would be incurred by about 60 additional hospitals with more than 25 medicine beds who have not yet implemented STRIDE. We used national median salary rates to estimate an expected budget for implementation and compared resource use across the two implementation strategies. We also projected the number of veterans expected to participate in STRIDE using the reach rates observed in the hospital‐level analysis. We varied key parameters where there is substantial uncertainty in the estimates.

We projected the national budget expected under three implementation scenarios: REP‐only, REP+CONNECT, and a combination. The combination approach assumes half of hospitals utilize each strategy, recognizing that the VA may choose to allow hospitals flexibility in their choice of implementation strategy. For the REP+CONNECT strategy, we applied mean REP and CONNECT implementation costs, including hospital and external team activities. In an exploratory analysis, we also compared possible costs for a self‐guided strategy excluding the costs for the external team facilitators, which will be evaluated in Implementing a Hospital‐Based Walking Program (STRIDE): Function QUERI 2.0 (STRIDE) (ClinicalTrials.gov Identifier: NCT04868656) and data collection is in progress.

We also projected national enrollment in STRIDE, using the reach rates observed in the hospital‐level analysis. Reach was defined as the percent of STRIDE‐eligible hospitalizations that were enrolled in STRIDE. For the hospital‐level analysis, reach was defined as the mean enrolled per month divided by mean eligible hospitalizations per month, where month was the number of postlaunch months observed, which varied by hospital. To estimate the potential reach in the national analysis, we obtained the ADC for general medicine across all VA facilities nationally (ADC = 5235) for the FY 2022 Quarter 1, which included STRIDE‐eligible and non‐eligible hospitalizations. We estimated the number of STRIDE‐eligible hospitalizations using the rate of eligible hospitalizations (per month) compared with the ADC among the eight study hospitals. For the base case, we estimated the national STRIDE‐eligible hospitalizations per month to be 1.2 times ADC which was the ratio of monthly eligible‐to‐ADC we observed in the REP+CONNECT group of study hospitals. Due to the high variation among study hospitals, we selected the lower, more conservative value to prevent overestimating the potentially eligible population nationally.

## 6 RESULTS

Participating hospitals represented six different census divisions (Table 1). Most facilities had a hospital 5‐star performance rating of 3 (five of eight) and facility complexity level of 1a, which indicates the highest acuity case mix and level of services (five of eight).^21^, ^22^ The ADC ranged from 32 to 120, with an average of 88 in the REP‐only group and 67 in the REP+CONNECT group. The STRIDE‐eligible hospitalizations (standardized per postlaunch month) were 165% of general medicine ADC for REP‐only (range 83%–248%) and 120% of ADC for REP+CONNECT (range 62%–161%) hospitals.

**TABLE 1:** Comparing STRIDE implementation costs (2022 USD) by hospital and implementation strategy (REP‐only and REP+CONNECT).

Hospital	REP‐only group					REP+CONNECT group				
	1	2	3	4	Mean	5	6	7	8	Mean
# months in post‐period	18	15	6	3	10.5	18	15	6	3	10.5
Hospital average daily census^a^	90	99	44	120	88.1	62	32	76	96	66.6
Hospital 5‐star performance rating^b^	3	5	3	4	3.8	3	3	3	4	3.3
Facility complexity level^c^	1a	1a	1b	1a	n/a	1b	1c	1a	1a	n/a
Census division	South Atlantic	West North Central	Middle Atlantic	West South Central	n/a	East South Central	West North Central	East North Central	South Atlantic	n/a
REP‐only costs (2022 USD)										
Hospital staff activities, #	86	71	27	53	59	51	53	81	60	61
Labor cost (Hospital), $	6309	4458	1910	3668	4086	3501	2780	3975	2299	3139
Labor cost (Durham), $	2177	2116	619	1230	1536	1824	1931	1551	1343	1662
Cost of REP‐only	8486	6574	2529	4898	5622	5325	4711	5526	3642	4801
CONNECT costs (2022 USD)										
Hospital staff activities, #						128	53	51	64	74
Labor cost (hospital), $						3689	1680	1768	1825	2240
Labor cost (Durham), $						2409	2409	2409	2409	2409
Cost of CONNECT	n/a	n/a	n/a	n/a	n/a	6098	4089	4177	4234	4649
Total implementation cost	8486	6574	2529	4898	5622	11,423	8800	9703	7876	9450

Abbreviations: REP, Replicating Effective Programs; STRIDE, A**S**sis**T**ed Ea**R**ly Mob**I**lization for hospitalize**D** older V**E**terans; USD, United States Dollars.

^a^ VHA facilities average daily census for Internal Medicine Beds FY22Q1.

^b^ US Department of Veterans Affairs (VFA) End‐of‐year Hospital 5‐star rating (1–5) indicates a VA hospital's quality of care relative to other VA hospitals and is based on data such as death rates, nursing turnover, patient satisfaction, and efficiency.

^c^ Facility complexity level classifies VHA facilities at levels 1a, 1b, 1c, 2, or 3 with level 1a being the most complex and level 3 being the least complex. The model is reviewed and updated with current data every 3 years. The peer grouping system is based on seven variables relating to patient population, clinical services complexity, and education and research. VHA Office of Productivity, Efficiency and Staffing (OPES). US Department of Veterans Affairs.

### 6.1 Cost of implementation activities

On average per hospital, 60 staff activities were tracked in REP, and their time spent resulted in a cost of $3193 (Table 1). External staff time resulted in a cost of $1439 per hospital, for a total of $4632 on REP activities per hospital. However, wide variation exists across hospitals, ranging from $2529 to $8486. On average, 74 hospital staff activities were tracked in CONNECT per hospital, and their time spent resulted in a cost of $2240. External staff time for CONNECT resulted in a cost of $2409 per hospital, for a total of $4649 spent on CONNECT activities per hospital. Less variation existed across hospitals, with CONNECT costs ranging from $4089 to $6098. Combining all implementation activities, the average implementation costs were $9450 in REP+CONNECT and $5622 in REP‐only strategy.

### 6.2 Cost of program delivery activities

**FIGURE 1:**
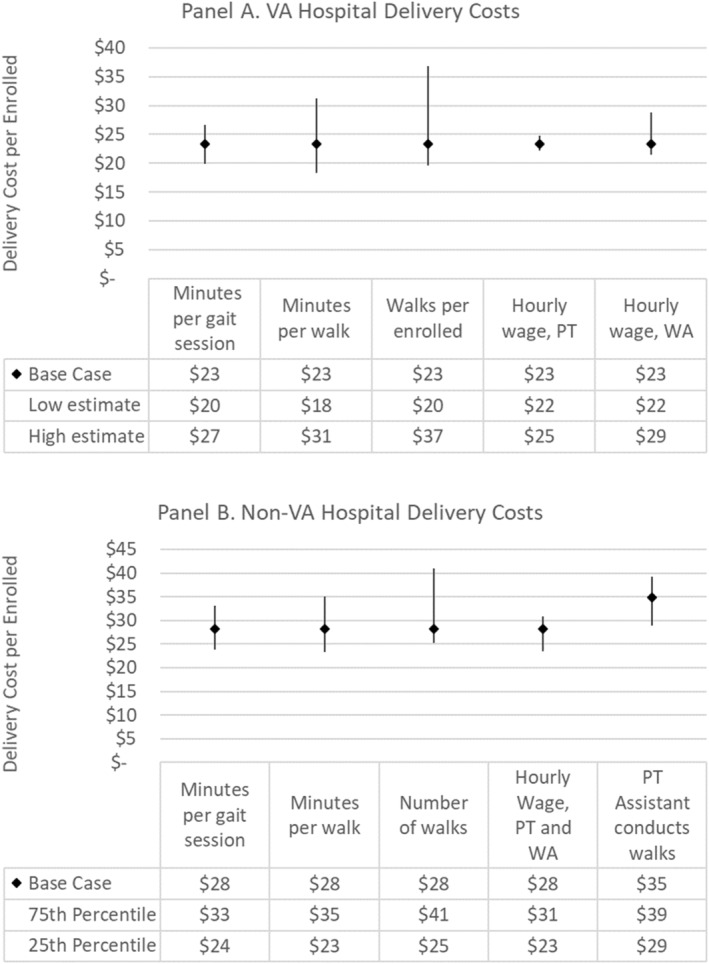
(A, B) One‐way sensitivity analysis to assess how variation in hospital implementation affects STRIDE program delivery costs per enrolled hospitalization (2022 USD). PT, Physical Therapist; STRIDE, A**S**sis**T**ed Ea**R**ly Mob**I**lization for hospitalize**D** older V**E**terans; USD, United States Dollars; VA, Veterans Affairs; WA, Walk Assistant.

Differences in wage rates and time per walk session (16.7 compared with 11.9 min) resulted in cost differences between the two implementation strategies, with an average of $31.5 and $20.6 per enrolled on average in the REP‐only and REP+CONNECT groups, respectively (Table 2). In non‐VA hospitals, base case cost was estimated at $28 per enrolled (Figure 1). Cost per gait assessment session ranged from $10.7 to $18.8 and cost per non‐gait session ranges from $2.4 to $11.4. The number of gait and walk sessions varied (1–5 total per enrolled) and contributed to cost differences between sites; though the means were similar in both implementation strategy groups.

**TABLE 2:** Total cost of STRIDE program delivery activities by hospital and implementation strategy.

Hospital	REP‐only group					REP+CONNECT group				
	1	2	3	4	Mean	5	6	7	8	Mean
STRIDE enrollment (standardized per month)										
Mean eligible hospitalizations	130.4	181.2	108.7	100.3	130.2	99.7	39.1	47.3	128.7	78.7
Mean enrolled participants	3.3	5.5	0.7	9.0	4.6	12.4	8.9	4.5	6.0	8.0
% of eligible hospitalizations enrolled	2.5%	3.0%	0.6%	9.0%	3.8%	12.5%	22.7%	9.5%	4.7%	12.3%
Initial gait assessments										
Minutes per gait assessment	23.5	22.6	16.2	17.7	20.0	14.6	19.2	22.7	14.2	17.7
Fringe benefits (+33%), $	1.3	1.3	1.3	1.3	1.3	1.3	1.3	1.3	1.3	1.3
Hourly rate for gait assessment, $	35.9	35.9	29.8	35.9	34.4	35.9	35.9	35.9	35.9	35.9
Cost per gait session, $	18.8	18.0	10.7	14.1	15.4	11.7	15.3	18.1	11.3	14.1
STRIDE walk session										
Minutes per session^a^	16.7	25.8	7.0	17.3	16.7	12.3	9.7	14.9	10.7	11.9
Fringe benefits (+33%), $	1.3	1.3	1.3	1.3	1.3	1.3	1.3	1.3	1.3	1.3
Hourly rate for walk assistant, $	15.6	20.3	15.6	29.8	20.3	15.6	20.3	15.6	20.3	17.9
Cost per walk session, $	5.8	11.6	2.4	11.4	7.8	4.3	4.3	5.2	4.8	4.6
Walk sessions per enrolled	2.8	4.8	1.0	1.8	2.6	2.7	3.8	1.6	1.7	2.5
Annualized total costs (2022 USD)										
Total delivery cost (annualized)	1088.9	4103.9	85.9	2521.5	1950.1	2673.6	2936.2	1090.8	1044.6	1936.3
Annualized enrollment	39.3	65.6	8.0	108.0	55.2	149.3	106.4	54.0	72.0	95.4
Delivery cost per enrolled participant	29.0	62.6	10.7	23.4	31.5	19.1	27.6	21.2	14.5	20.6

Abbreviations: REP, Replicating Effective Programs; STRIDE, A**S**sis**T**ed Ea**R**ly Mob**I**lization for hospitalize**D** older V**E**terans; USD, United States Dollars.

^a^ Walk times include the walk time documented in the health record plus the survey‐reported estimates for minutes spent before and after the actual walk.

In the one‐way sensitivity analyses, cost per participant was driven by variation in the number of walks and minutes per walk per hospital (Figure 1). The base case, high and low values for the scenario analyses (Table 3) were intended to represent a range of likely input values based on the data from the study sites. Holding other input parameters at their base case values, the observed minimum number of walks resulted in a program delivery cost of $20 per participant compared with $37.0 for the highest observed number of walks. The total minutes spent on a STRIDE walk session had the second highest difference between low and high values, at a cost of $18 and $31.

**TABLE 3:** Assumptions for estimating VA hospital and non‐VA hospital costs to support STRIDE program delivery annually (2022 USD).

	Base case	Low	High
Enrollment factors			
National mean staffed hospital beds (Medicare Hospital cost reports 2022)	130	100	150
Occupancy rate (Centers for Disease Control 2022)	75%	60%	85%
Study eligibility rate (eligible hospitalizations per month/ADC)	1.2	1.0	1.5
Study penetration rate (enrolled hospitalizations/eligible hospitalizations)	8.1%	2.6%	15.8%
VA labor costs (general schedule pay scales 2022)			
Hourly rate for gait assessment (physical therapist)	$35	$30	$36
Hourly rate for walk assistant (nurse assistant)	$19	$16	$30
Fringe benefits (+33%)	1.3	1.3	1.3
Non‐VA labor costs (Bureau of Labor Statistics 2022)			
Hourly rate for gait assessment (physical therapist)	$47	$39	$52
Hourly rate for walk assistant (nurse assistant)	$17	$15	$19
Hourly rate for walk assistant (physical therapy assistant)	$31	$26	$36
Fringe benefits (+33%)	1.3	1.3	1.3
Gait assessment sessions			
Total minutes per session	18.8	14.2	23.5
Cost of one gait session	$20	$12	$27
Number of gait assessments per enrolled hospitalization	1.0	1.0	1.0
Non‐gait walk sessions			
Total minutes per session	14.3	7.0	25.8
Cost of one non‐gait walk	$6	$2	$11
Number of non‐gait walks per enrolled hospitalization^a^	1.5	1.0	3.8

Abbreviations: ADC, average daily census, estimated for non‐VA hospitals as staffed hospital beds times occupancy rate; REP, Replicating Effective Programs; STRIDE, A**S**sis**T**ed Ea**R**ly Mob**I**lization for hospitalize**D** older V**E**terans; USD, United States Dollars.

^a^ Enrolled hospitalizations per hospital per year is estimated as the number of eligible hospitalizations (study eligibility rate*ADC*12) times the study penetration rate (enrolled hospitalizations/eligible hospitalizations).

### 6.3 National budget projections

**FIGURE 2:**
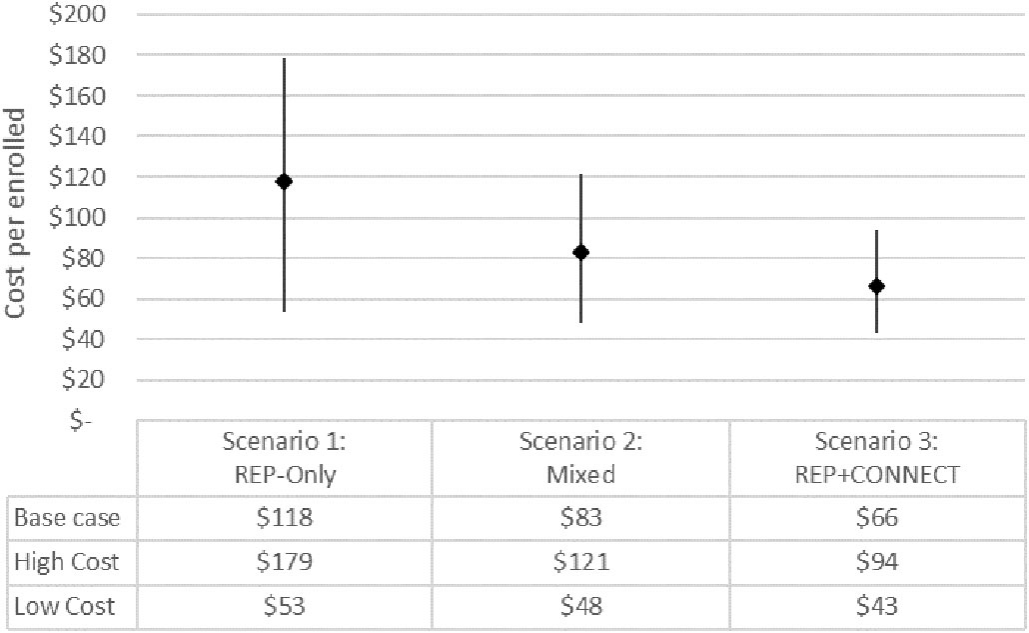
Scenario analyses estimating first‐year STRIDE implementation costs per enrolled veteran and uncertainty by national implementation strategy (2022 USD). REP, Replicating Effective Programs; STRIDE, A**S**sis**T**ed Ea**R**ly Mob**I**lization for hospitalize**D** older V**E**terans; USD, United States Dollars. The scenario analysis uses the high and low‐cost estimates while holding enrollment numbers constant at the base case estimate. Results varying enrollment can be found in Table S2.

Program participation rate was the key driver of variation in program delivery costs. Observed participation rates (percentage of eligible hospitalizations enrolled per month) varied across hospitals from 0.6% to 22.7% with a mean of 8.1%. On average, participation was higher in the REP+CONNECT group (12.3%) than in the REP‐only group (3.8%). In the base case, we estimated annual national STRIDE enrollment to be 6071, which assumed an 8.1% participation rate among 75,384 eligible hospitalizations nationally (Appendix B). At an average delivery cost of $23.3 per participant, enrollment of 6071 would require a national budget of $141,533 for program delivery. A 15.8% reach rate would require $277,918, and a 2.6% reach rate would require $46,367 to deliver STRIDE nationally. In scenario analyses, we estimated potential national budgets to support different implementation strategies (Figure 2). In the REP‐only scenario, the base case cost estimate was $337,305, with a low of $151,740 and a high of $509,160; the base case enrollment estimate was 2848 STRIDE participants, with a low of 462 and a high of 6762 in the first year; and the implementation cost per enrolled participant was $118 in the base case, with a low of $53 and a high of $179. In the REP+CONNECT scenario, the base case cost estimate was $616,273, with a low of $397,063 and a high of $875,031; the base case enrollment estimate was 9294 STRIDE participants, with a low of 3515 and a high of 17,080 in the first year; and the implementation cost per enrolled was $66 in the base case, with a low of $43 and a high of $94.

In exploratory projections, we assumed 30 hospitals used a self‐guided implementation strategy and 30 hospitals used a REP‐only facilitated implementation strategy and estimated a budget projection of $291,240. We were unable to estimate enrollment due to lack of reach data among hospitals using self‐guided implementation.

## 7 DISCUSSION

We conducted a BIA to assess the resources needed to implement STRIDE in inpatient settings for the first year. We found that STRIDE is a low‐cost intervention to deliver in VA and non‐VA settings ($23 and $28 per enrolled, respectively), and program enrollment has the biggest impact on the resources needed for delivering STRIDE. In the first year, implementation costs are likely to exceed the program delivery costs and require substantial investment (staff time/resources) from the hospital for training and implementation. Subsequent to the studies already demonstrating effectiveness in the VA, this BIA is intended to inform the business case for disseminating STRIDE nationally within the VA health system. We were unable to identify any other economic evaluations of mobility interventions in VA, non‐VA, or international settings; however, we found one published protocol that proposes to evaluate delivery costs for an inpatient assisted ambulation program.^23^ Thus, this novel evidence about the resources necessary to deliver STRIDE may be useful for non‐VA health systems seeking to improve mobility and patient outcomes.

The Function QUERI team disseminated STRIDE results with VA operational partners through two strategies.^4^, ^6^ First, we communicated quantitative results through multiple briefings and presentations within the VA. For example, this analysis was presented to the STRIDE diffusion network, which is a national peer network of VA clinicians and managers to support implementation of STRIDE local hospitals. Second, we facilitated deeper discussion around the interpretation and application of the findings with an advisory group of national VA program leaders representing Physical Medicine Rehabilitation, Office of Nursing Services, Geriatrics and Extended Care services. The advisory group was tasked with planning the transition from a research study to a clinical operations infrastructure. With these partners, we are making a plan to transition the program over to one of these operational partners from clinical implementation.

Additionally, STRIDE is aligned with VA's Whole Health transformation, which seeks to move beyond disease‐oriented care to focus on promoting well‐being from the person's perspective and beliefs among both older patients and hospital providers that “mobility is medicine.”^24^, ^25^, ^26^, ^27^, ^28^ In 2022, 59 hospitals were planning to implement STRIDE (including those already implemented as a part of the trial). Ongoing work will evaluate the broad scale implementation of STRIDE at 35 hospitals as a part of the second round of funding of the Function QUERI project grant (started in 2021).^29^ In this trial, in a lower intensity REP approach, hospitals will independently use low‐touch resources (e.g., toolkits and recorded webinars) to prepare for implementation and have access to monthly office hours with external facilitators.^30^ The new project will collect data on the hospitals utilizing low‐touch implementation to provide further guidance on the most efficient and effective implementation strategies for expanding STRIDE and other evidence‐based programs.

The type of implementation strategy, REP or REP+CONNECT, may impact the enrollment rates of the program among eligible veterans; however, we do not know if the differential enrollment across implementation strategies observed at the hospital level would translate to the national experience. If the national experience is consistent with the 8‐hospital study data, we would expect implementation costs in the first year to be greater than the delivery costs. The most resource intensive implementation strategy, REP+CONNECT, was implemented among the hospitals that also had the highest reach rate among eligible hospitalizations. More evidence is needed to understand the causal relationship between intensity of implementation support and enrollment. Furthermore, understanding the mechanisms for increasing reach, and therefore the population outcomes, is critical for informing efficient allocation of resources for implementation. After the first year, the program delivery expenses would encompass nearly all of the budget, though there may be some need for internal training and maintenance of best practices to sustain strong enrollment and outcomes.

Hospitals participating in the Function QUERI STRIDE study invested substantially in the implementation training activities. A high number of staff participated across a wide range of roles, and their time is valuable. Hospital resource use contributed 70% of REP costs and 50% of CONNECT costs. We also found that in the 8‐hospital study, hospital resources dedicated to REP activities were lower among the hospitals participating in CONNECT than the hospitals participating in REP‐only. It is possible that limited hospital capacity required reducing REP time to accommodate time for CONNECT or that there was some overlap between the supports provided by the two implementation strategies, or that some efficiency was gained by combining the two strategies. While we measure the impact of salaries on the budget estimates, we do not account for the opportunity cost that might be present if their time had been invested in directly addressing veteran patients' needs. However, we expect that the hospital administrative leadership would always put patients' needs first and make decisions for participation in trainings appropriately.

This descriptive study presents potential national scenarios based on data from eight hospitals to inform VA decision‐making around resource allocation and implementation facilitation. Given the variability in the eight study hospitals outcomes, we do not know if the mean effects observed at the hospital level would translate to the national experience or to non‐VA contexts. We do not know if the observed reach among the different implementation strategy groups was due to the implementation strategy or other factors about the hospitals that were randomized to that strategy. Due to the small number of hospitals (4) in each group, we are unable to assess the causal relationship. VA facilities may use a special pay authority (Title 38) to recruit and retain physicians and match market wages. In this study, cost estimates used VA general scale wages rather than actual wages which are impacted by Title 38; however, since physician roles were not involved in the STRIDE delivery, this would have a small impact on the projected VA budget estimates. While some studies have found reductions in use of facility‐based services, there is a need for stronger evidence around the downstream effects of STRIDE and other mobility programs on resource use across care settings. Despite this limitation, the potential for savings compared with the relatively low cost of the intervention makes it very likely the intervention will be cost‐effective and cost‐saving in some populations.

## 8 CONCLUSION

This early evaluation provides practical information about the financial resources needed to deliver STRIDE and support the expansion of mobility programs in and out of the VA health system. The budget impact evaluation compares costs for different intensity of implementation strategies to inform the business case for disseminating STRIDE nationally within the VA. With the right resources, VA could improve outcomes for hospitalized older veterans through wider implementation of the STRIDE mobility program.

## FUNDING INFORMATION

This work was funded by the United States (US) Department of Veterans Affairs Quality Enhancement Research Initiative (QUE‐16‐170) and by the Center of Innovation to Accelerate Discovery and Practice Transformation (CIN 13‐410) at the Durham VA Health Care System. The funding agency had no role in the design or conduct of the study; collection, analysis, or interpretation of the data; or preparation, review, or approval of the manuscript. The contents do not represent the views of the US Department of Veterans Affairs or the US Government.

## CONFLICT OF INTEREST STATEMENT

The authors declare no conflicts of interest.

## Supporting information


**Appendix A:** Supporting information.

**Appendix B:** Supporting information.
