# Supplementary material for: The business case for hospital mobility programs in the veterans health care system: Results from multi‐hospital implementation of the STRIDE program
Source: Health Serv Res. 2024 Apr 17;59(Suppl 2):e14307. doi: 10.1111/1475-6773.14307 (PMC11540580; doi:10.1111/1475-6773.14307)
Supplement: Supplementary file 1 — Appendix A: Supporting information. [file HESR-59-0-s001.docx]

**Time Study Data Collection Form (STRIDE)**

Site: *____________* Role (staff completing form): _______________

*Instructions: Choose 3 patients and then observe the time you spend completing the STRIDE activity.*

**Patient 1** Date of Completion: __________ Ward: ______ Activity (circle): Gait Assessment or Daily Walk

| **Question** | **Response** |
| --- | --- |
| 1. Minutes spent to find a patient to walk (clock starts when you enter patient room): |  |
| 1. Minutes spent preparing patient for STRIDE activity (in the room, reviewing chart, getting patient of out bed, etc.): |  |
| 1. Minutes spent completing the STRIDE activity with the patient (once they are out of bed): |  |
| 1. Minutes spent after the STRIDE activity (documentation): |  |
| 1. Miscellaneous time (walk attempts, coordination with PACT team, etc… write activity and time in minutes): |  |
| 1. How much assistance was needed for this patient to get out of bed and complete STRIDE activity? | Minimum Moderate Maximum |

**Patient 2** Date of Completion: __________ Ward: ______ Activity (circle): Gait Assessment or Daily Walk

| **Question** | **Response** |
| --- | --- |
| 1. Minutes spent to find a patient to walk (clock starts when you enter patient room): |  |
| 1. Minutes spent preparing patient for STRIDE activity (in the room, reviewing chart, getting patient of out bed, etc.): |  |
| 1. Minutes spent completing the STRIDE activity with the patient (once they are out of bed): |  |
| 1. Minutes spent after the STRIDE activity (documentation): |  |
| 1. Miscellaneous time (walk attempts, coordination with PACT team, etc… write activity and time in minutes): |  |
| 1. How much assistance was needed for this patient to get out of bed and complete STRIDE activity? | Minimum Moderate Maximum |

**Patient 3** Date of Completion: __________ Ward: ______ Activity (circle): Gait Assessment or Daily Walk

| **Question** | **Response** |
| --- | --- |
| 1. Minutes spent to find a patient to walk (clock starts when you enter patient room): |  |
| 1. Minutes spent preparing patient for STRIDE activity (in the room, reviewing chart, getting patient of out bed, etc.): |  |
| 1. Minutes spent completing the STRIDE activity with the patient (once they are out of bed): |  |
| 1. Minutes spent after the STRIDE activity (documentation): |  |
| 1. Miscellaneous time (walk attempts, coordination with PACT team, etc… write activity and time in minutes): |  |
| 1. How much assistance was needed for this patient to get out of bed and complete STRIDE activity? | Minimum Moderate Maximum |

*During this shift, could you please estimate the number of minutes you spend on other STRIDE-related activities (away from the patient)?*

| **Question** | **Response** | |
| --- | --- | --- |
| Triaging consults (if applicable) | __________________minutes OR N/A | |
| Recruitment time (identifying patients, coordinating with other providers, marketing the program, etc.):  Enter the number of minutes, or if not applicable, circle N/A. | _________________minutes OR N/A | |
| Other time spent on STRIDE related activities? Please describe the activity and time. | Activity | Time (minutes) |
|  |  |  |

*Please include information about equipment used and/or purchased to deliver STRIDE:*

| **Question** | **Response** |
| --- | --- |
| Have you used any equipment for STRIDE patients? (circle one) | Yes No |
| If yes, what type of equipment was used? |  |
| Did your team purchase equipment for STRIDE? (circle one) | Yes No Don’t know |
| If yes, what equipment did your team purchase? | [List Here] or Don’t know |
| If yes, what was the funding source? | [List Here] or Don’t know |
| If no, do you need equipment to deliver STRIDE? (circle one) | Yes No |
