# Supplementary material for: The business case for hospital mobility programs in the veterans health care system: Results from multi‐hospital implementation of the STRIDE program
Source: Health Serv Res. 2024 Apr 17;59(Suppl 2):e14307. doi: 10.1111/1475-6773.14307 (PMC11540580; doi:10.1111/1475-6773.14307)
Supplement: Supplementary file 2 — Appendix B: Supporting information. [file HESR-59-0-s002.docx]

**Appendix B: Supplemental Tables**

**Table S1. Equipment purchased for the sole hospital that reported purchasing equipment for STRIDE Implementation in the VA 8-site implementation study**

| **EQUIPMENT (for years 1 and 2)** | **Cost/unit** | **# units** | **Total Cost** |
| --- | --- | --- | --- |
| Rolling walkers | $34 | 8 | $272 |
| Tape measures | $10 | 5 | $50 |
| Stop watch | $35 | 5 | $175 |
| Pedometers |  |  |  |
| Distance Markers |  |  |  |
| Portable pulse oximeters | $78 | 8 | $624 |
| Gait belts | $38 | 8 | $304 |
| Rollators | $190 | 8 | $1,520 |
| Canes | $18 | 8 | $144 |
| **Total Equipment Costs** |  |  | **$3,089** |

**Table S2. Projected eligible hospitalizations and reach estimates used for national projections (first year of implementation)**

|  | **Base Case** | **Low** | **High** |
| --- | --- | --- | --- |
| # Eligible hospitalizations* | 75,384 | 62,820 | 94,230 |
| Reach ( % of Eligible Enrolled) |  |  |  |
| Overall | 8.1% | 2.6% | 15.8% |
| REP+CONNECT | 12.3% | 4.7% | 22.7% |
| REP-only | 3.8% | 0.6% | 9.0% |

**Eligible hospitalizations per year are estimated as (Study eligibility rate*ADC*12). Hospital number of enrolled hospitalization per year is estimated by taking the hospital number of eligible hospitalizations per year times the study penetration rate (Enrolled hospitalizations in the study /eligible hospitalizations in the study).*

**Table S3. Base case and extreme values used in estimating national first-year implementation cost projections (2022 USD)**

| **Implementation Costs Per Hospital (first year)** | **Base Case** | **Low** | **High** |
| --- | --- | --- | --- |
| *REP Only Costs (among 4 REP only hospitals)* |  |  |  |
| Cost of implementation team (Hospital) | 4,086.25 | 1,910.00 | 6,309.00 |
| Cost of implementation team (Durham) | 1,535.50 | 619.00 | 2,177.00 |
| **Cost of REP only** | **5,621.75** | **2,529.00** | **8,486.00** |
| *CONNECT Costs (among 4 REP+CONNECT hospitals)* |  |  |  |
| Cost of implementation team (Hospital) | 2,240.49 | 1,679.73 | 3,688.87 |
| Cost of implementation team (Durham) | 2,408.98 | 2,408.98 | 2,408.98 |
| Cost of CONNECT | 4,649.47 | 4,088.71 | 6,097.85 |
| **Total Implementation Cost (REP+CONNECT)** | **10,271.22** | **6,617.71** | **14,583.85** |

**Table S4. Scenario analyses estimating national cost (2022 USD) and reach for different implementation strategies**

| *Year 1 Scenario Projections* | **Scenario 1**  **REP-ONLY** | **Scenario 2**  **Mixed** | | **Scenario 3**  **REP+CONNECT** |
| --- | --- | --- | --- | --- |
| **National implementation cost, S** | *60 hospitals* | *30 REP-only &*  *30 REP+ CONNECT* | | *60 sites* |
| Base case estimate | 337,305 | 476,789 | | 616,273 |
| High case estimate | 509,160 | 692,096 | | 875,031 |
| Low case estimate | 151,740 | 274,401 | | 397,063 |
| **National projected enrollment*** | | |  | |
| Base reach estimate | 2,848 | 5,713 | | 9,294 |
| High reach estimate | 6,762 | 11,921 | | 17,080 |
| Low reach estimate | 462 | 1,989 | | 3,515 |
| **Implementation cost per enrolled, $** | | |  | |
| Base case estimate | 118 | 83 | | 66 |
| High cost, High enrollment | 75 | 58 | | 51 |
| Low cost, low enrollment | 328 | 138 | | 113 |
| *Reach rate from site level analysis multiplied by 75,384 eligible hospitalizations annually across 60 sites | | | | |
